# Modulation of mitochondrial activity by sugarcane (*Saccharum officinarum* L.) top extract and its bioactive polyphenols: a comprehensive transcriptomics analysis in C2C12 myotubes and HepG2 hepatocytes

**DOI:** 10.1007/s13659-023-00423-x

**Published:** 2024-01-05

**Authors:** Kengo Iwata, Farhana Ferdousi, Yoshinobu Arai, Hiroko Isoda

**Affiliations:** 1 Alliance for Research on the Mediterranean and North Africa (ARENA), University of Tsukuba, Tsukuba, Ibaraki 305-8572 Japan; 2 Nippo Co., Ltd., Daito, Osaka 574-0062 Japan; 3 Institute of Life and Environmental Sciences, University of Tsukuba, Tsukuba, Ibaraki 305-8572 Japan; 4 AIST-University of Tsukuba Open Innovation Laboratory for Food and Medicinal Resource Engineering (FoodMed-OIL), Tsukuba, Ibaraki 305-8572 Japan

**Keywords:** Sugarcane top, Polyphenol, Mitochondria, PGC-1α, Fatty acid metabolism, Inflammatory cytokine

## Abstract

Age-related mitochondrial dysfunction leads to defects in cellular energy metabolism and oxidative stress defense systems, which can contribute to tissue damage and disease development. Among the key regulators responsible for mitochondrial quality control, peroxisome proliferator-activated receptor gamma coactivator 1-alpha (PGC-1α) is an important target for mitochondrial dysfunction. We have previously reported that bioactive polyphenols extracted from sugarcane top (ST) ethanol extract (STEE) could activate neuronal energy metabolism and increase astrocyte PGC-1α transcript levels. However, their potential impact on the mitochondria activity in muscle and liver cells has not yet been investigated. To address this gap, our current study examined the effects of STEE and its polyphenols on cultured myotubes and hepatocytes in vitro. Rhodamine 123 assay revealed that the treatment with STEE and its polyphenols resulted in an increase in mitochondrial membrane potential in C2C12 myotubes. Furthermore, a comprehensive examination of gene expression patterns through transcriptome-wide microarray analysis indicated that STEE altered gene expressions related to mitochondrial functions, fatty acid metabolism, inflammatory cytokines, mitogen-activated protein kinase (MAPK) signaling, and cAMP signaling in both C2C12 myotubes and HepG2 hepatocytes. Additionally, protein–protein interaction analysis identified the PGC-1α interactive-transcription factors-targeted regulatory network of the genes regulated by STEE, and the quantitative polymerase chain reaction results confirmed that STEE and its polyphenols upregulated the transcript levels of PGC-1α in both C2C12 and HepG2 cells. These findings collectively suggest the potential beneficial effects of STEE on muscle and liver tissues and offer novel insights into the potential nutraceutical applications of this material.

**Graphical Abstract:**

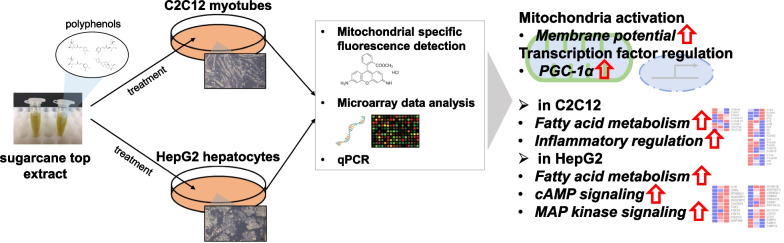

**Supplementary Information:**

The online version contains supplementary material available at 10.1007/s13659-023-00423-x.

## Introduction

Mitochondria, often referred to as the cellular "powerhouses" responsible for ATP production, play a crucial role in maintaining cellular function and appropriate responses to stress. In non-mitotic cells like cardiomyocytes and skeletal muscle cells, the aging process incites mitochondrial dysfunction, which subsequently triggers the activation of the DNA damage response and the innate immune response, thereby leading to chronic inflammation [1]. The accumulation of mitochondrial DNA (mtDNA) mutations and alterations in mitochondrial quality control mechanisms, such as the balance between mitochondrial biosynthesis and mitophagy, as well as the balance between mitochondrial fusion and fission, are considered contributing factors to age-related mitochondrial dysfunction [2–4].

The peroxisome proliferator-activated receptor gamma coactivator 1-alpha (PGC-1α) is one of the key regulators of mitochondrial quality control [5–7]. PGC-1α induces expression of its downstream effector transcription factor A mitochondrial (TFAM) involved in mtDNA maintenance and transcription, and promotes mitochondrial biogenesis. PGC-1α has also been reported to regulate mitophagy with other effectors [8, 9]. In addition, molecular pathways such as the AMP-activated protein kinase (AMPK) and mitogen-activated protein kinase (MAPK) pathways have been reported to regulate both mitochondrial biogenesis and mitophagy [10–12].

We have previously reported the identification of the four major polyphenolic components in sugarcane top (ST) ethanolic extract (STEE), namely, 3-*O*-caffeoylquinic acid (3CQA), 5-*O*-caffeoylquinic acid (5CQA), 3-*O*-feruloylquinic acid (3FQA), and Isoorientin (ISO, chemically luteolin-6-C-glucoside). We also demonstrated that STEE could ameliorate spatial learning and memory deficits in senescence-accelerated model mice via promoting energy metabolism and neurogenesis in vivo [13]. Furthermore, we extended our investigation to unveil that the coordinated action of 3CQA, 5CQA, and ISO within STEE elevated PGC-1α transcription levels in immature astrocytes, promoting their branching morphology in vitro [14]. These findings encourage us to investigate the effects of STEE and its bioactive polyphenols on metabolically active muscle and liver functions. It is well-documented that mitochondrial dysfunction and the associated rise in oxidative stress significantly contribute to the decline in muscle mass and function, as well as the onset of liver inflammation and fibrosis [15–18]. Previous studies have indicated that CQA stimulates glucose transport in skeletal muscle in vivo and enhances glycolytic and electron transport systems in HepG2 hepatocytes in vitro [19, 20]. Also, ISO has been reported to protect against oxidative damage in both C2C12 myotubes and HepG2 hepatocytes in vitro, through regulating the genes related to mitochondrial function [21–23]. However, the synergistic functional effects of these compounds on the mitochondrial functions of myotubes and hepatocytes have not yet been investigated.

We, therefore, aimed herein to obtain the data concerning the alterations induced by STEE and its polyphenols in cultured myotubes and hepatocytes in vitro, with a specific emphasis on metabolic processes and mitochondrial function, in order to uncover their unexplored functional attributes. We conducted comprehensive transcriptome-wide analyses using microarray technology, which provided valuable insights into the biological and molecular changes by STEE and its polyphenols.

## Results

### Six-hour exposure to STEE or mixed-compound treatment induced a significant increase in the mitochondrial membrane potential of C2C12 myotubes

First, we tested the effect of STEE on cell viability using the 3-(4,5-Dimethylthiazol-2-yl)-2,5-Diphenyltetrazolium Bromide (MTT) assay. In differentiated C2C12 myotubes, treatment with STEE for 24 or 48 h did not yield any significant changes in absorbance across various concentrations. Similarly, in HepG2 hepatocytes, exposure to STEE for either 24 or 48 h exhibited no significant changes in absorbance at any concentration (Additional file 1: Fig. S1). These results suggest that STEE does not affect the viability of either C2C12 myotubes or HepG2 hepatocytes in vitro within the concentration range used in this study.

**Fig. 1:**
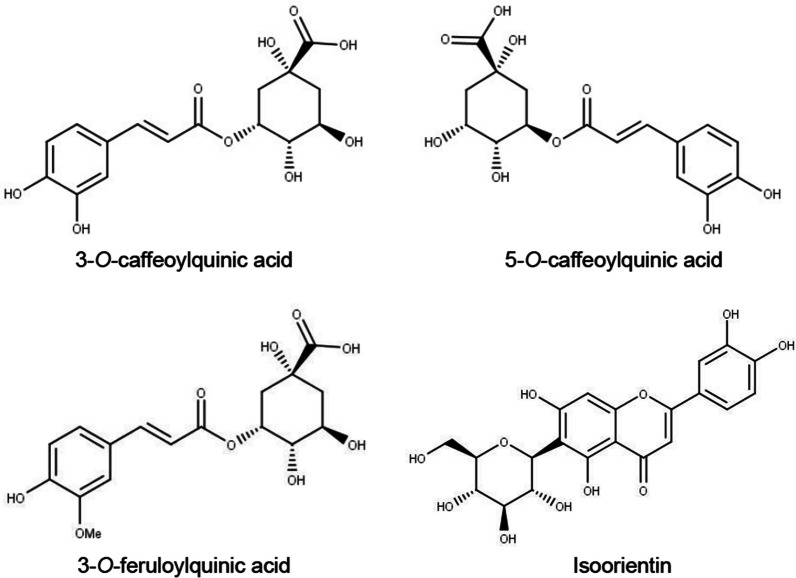
Structures of the polyphenolic components of sugarcane top extract

Considering the results, we opted for a concentration of 50 µg/mL of STEE for the subsequent Rhodamine 123 (Rh123) assay. Also, we treated the cells with polyphenols of STEE either individually or in combination. The concentrations were calibrated to match the amounts contained within the STEE (for example, 3CQA = 0.50 µM; 5CQA = 0.70 µM; 3FQA = 0.85 µM; ISO = 0.48 µM were equivalent to 50 µg/mL) as previously described [13, 14]. The structures of the compounds are shown in Fig. 1 and the combinations of the compounds are explained in Table 1.

**Table 1:** Combinations of the compounds used for the treatment in this study

Number of compounds	Combination	Concentration	Treatment no.
Two	3CQA + 5CQA	3CQA = 0.50 µM 5CQA = 0.70 µM	(1)
	3CQA + 3FQA	3CQA = 0.50 µM 3FQA = 0.85 µM	(2)
	3CQA + ISO	3CQA = 0.50 µM ISO = 0.48 µM	(3)
	5CQA + 3FQA	5CQA = 0.70 µM 3FQA = 0.85 µM	(4)
	5CQA + ISO	5CQA = 0.70 µM ISO = 0.48 µM	(5)
	3FQA + ISO	3FQA = 0.85 µM ISO = 0.48 µM	(6)
Three	3CQA + 5CQA + 3FQA	3CQA = 0.50 µM 5CQA = 0.70 µM 3FQA = 0.85 µM	(7)
	3CQA + 5CQA + ISO	3CQA = 0.50 µM 5CQA = 0.70 µM ISO = 0.48 µM	(8)
	3CQA + 3FQA + ISO	3CQA = 0.50 µM 3FQA = 0.85 µM ISO = 0.48 µM	(9)
	5CQA + 3FQA + ISO	5CQA = 0.70 µM 3FQA = 0.85 µM ISO = 0.48 µM	(10)

**Fig. 2:**
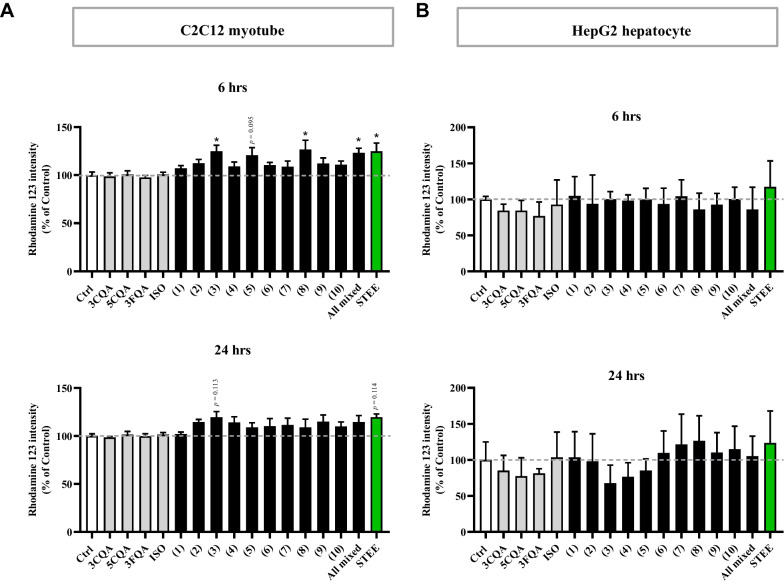
Fluorescence intensity of Rh123 in the cells. **A** After C2C12 myotubes were treated with STEE or its major constituents; 3CQA, 5CQA, 3FQA, or ISO for 6 or 24 h, the cells were stained with Rh123. One-way ANOVA followed by Dunnett's post hoc test was performed to assess statistical significance: * *p* < 0.05. **B** After HepG2 hepatocytes were treated with STEE or its major constituents; 3CQA, 5CQA, 3FQA, or ISO for 6 or 24 h, the cells were stained with Rh123. Comparisons were performed using Kruskal − Wallis test followed by Dunn's post hoc test. Results are expressed as relative percentages compared with the control (mean ± SEM, n = 3)

Treatment of STEE with C2C12 myotubes for 6 h significantly increased Rh123 intensity (Fig. 2A). Also, treatments designated as No. 3, 8, and 10, significantly increased Rh123 intensity in C2C12 myotubes (approximately 1.24-fold for each) at 6 h. Furthermore, treatment No. 5 over a 6 h period displayed a trend of increased fluorescence intensity (*p* = 0.095) (Fig. 2A). No significant changes in Rh123 intensity were observed in C2C12 myotubes following a 24 h treatment with any of the samples (Fig. 2A). These results suggest that the extract increased the mitochondrial membrane potential (MMP) of C2C12 myotubes during a 6 h of treatment period and this effect could be attributed to the synergistic action of 3CQA, 5CQA, and ISO present in the extract.

In the case of HepG2 cells, no significant changes in Rh123 intensity across any of the samples or treatment durations (Fig. 2B). Nevertheless, although not statistically significant, there was an approximate 1.25-fold increase in fluorescence intensity following 24 h treatment with STEE, No. 7, and 8. There appeared to be a trend toward greater fluctuations in fluorescence intensity after 24 h of treatment compared to the 6 h timeframe, suggesting that the effects of STEE and its polyphenols on MMP may exhibit variability depending on the duration of treatment.

### Transcriptomic profiling of STEE-treated myotubes and hepatocytes by microarray

To gain mechanistic insight into the effects of STEE, we performed the transcriptomic analysis of the C2C12 myotubes and HepG2 hepatocytes by microarray. The RNA samples extracted from nontreated control cells were compared with the RNA samples extracted from C2C12 myotubes subjected to two different concentrations of STEE for 6 h—30 µg/mL and 50 µg/mL (referred to as STEE30-M and STEE50-M, respectively). Likewise, the RNAs from nontreated HepG2 cells were compared with the RNAs obtained from cells exposed to two distinct concentrations of STEE for 24 h, namely 15 µg/mL and 30 µg/mL (referred to as STEE15-H and STEE30-H, respectively).

**Fig. 3:**
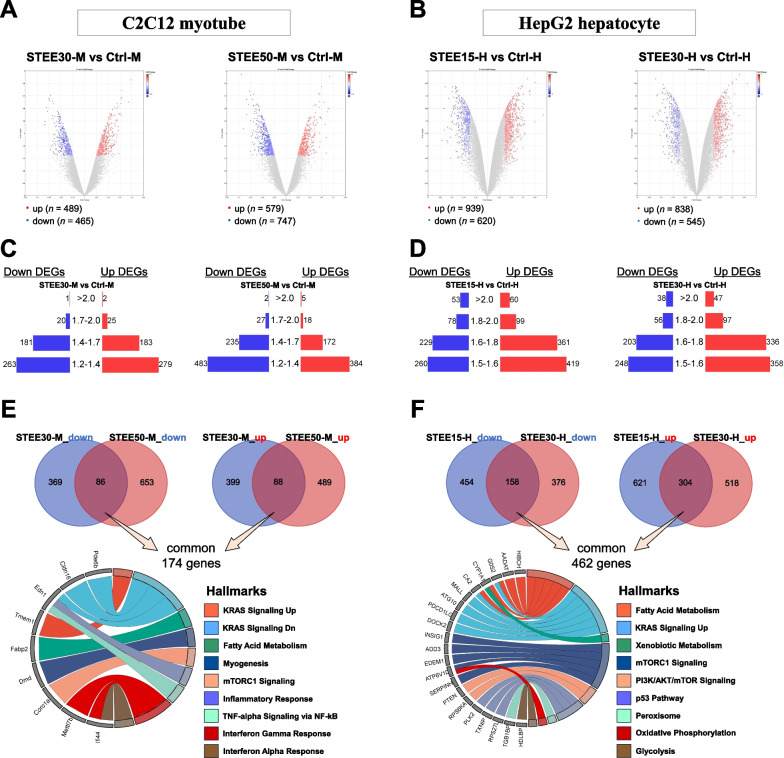
Microarray-identified gene expression profile reflecting STEE treatment on C2C12 and HepG2. Volcano plots depict DEGs between **A** STEE (lower and higher concentrations)-treated C2C12 myotubes and nontreated control, and **B** STEE (lower and higher concentrations)-treated HepG2 hepatocytes and nontreated control. DEGs satisfied with the efficiency (*p*-value < 0.05, above the log2-transformed fold change thresholds) are shown as colored dots. **C**, **D** The distribution of DEGs by fold-changes for each comparison is shown in the butterfly bar charts. **E** Venn diagrams showing overlapped and unique sets of DEGs between the groups. The blue circle denotes down- or up-regulated DEGs in STEE30-M compared to Ctrl-M, and the red circle denotes down- or up-regulated DEGs in STEE50-M compared to Ctrl-M. The code diagram displays the hallmark gene sets related to the 174 commonly overlapped DEGs. **F** The blue circle denotes down- or up-regulated DEGs in STEE15-H compared to Ctrl-H, and the red circle denotes down- or up-regulated DEGs in STEE30-H compared to Ctrl-H. The code diagram displays the hallmark gene sets related to the 462 commonly overlapped DEGs

In comparison to the control group, STEE30-M exhibited 954 differentially expressed genes (DEGs), with 489 being upregulated and 465 being downregulated (Fig. 3A). In the case of STEE50-M, there were 1326 DEGs, consisting of 579 upregulated and 747 downregulated genes (Fig. 3A). Similarly, in the case of STEE15-H, 1559 DEGs were detected, including 939 upregulated and 620 downregulated genes, in comparison to the control group (Fig. 3B). For STEE30-H, there were 1383 DEGs, with 838 genes upregulated and 545 genes downregulated (as represented in Fig. 3B). Compared to the control group, a greater number of genes exhibited downregulation than upregulation in STEE50-M. Furthermore, when comparing the STEE15-H group with the control, there were more DEGs than when comparing the STEE30-H group with the control. The distributions of fold change (FC) of the DEGs are shown in the butterfly charts (Fig. 3B for the C2C12 group; Fig. 3C for the HepG2 group).

We assessed the degree of overlap of DEGs in STEE30-M and STEE50-M. Among the DEGs that were downregulated compared to controls, 86 genes were common (Fig. 3E). Among the DEGs that were upregulated compared to controls, 88 genes were common (Fig. 3E). A total of 174 commonly regulated DEGs in STEE-treated C2C12 myotubes were related to the several biological states or processes categorized as the hallmark gene set as follows: Kras Signaling Dn (Systemic name: M5956), Kras Signaling Up (M5953), Fatty Acid Metabolism (M5935), Myogenesis (M5909), mTORC1 Signaling (M5924), Inflammatory Response (M5932), TNF-alpha Signalling Via NF-kB (M5890), Interferon Gamma Response (M5913), and Interferon Alpha Response (M5913) (Fig. 3E).

We also assessed the degree of overlap of DEGs in STEE15-H and STEE30-H. Among the DEGs that were downregulated compared to controls, 158 genes were common (Fig. 3F). Among the DEGs that were upregulated compared to controls, 304 genes were common (Fig. 3F). A total of 462 commonly regulated DEGs in STEE-treated HepG2 cells were related to the hallmark gene sets as follows: Fatty Acid Metabolism, Kras Signaling Up, Xenobiotic Metabolism (M5934), mTORC1 Signaling, PI3K/AKT/mTOR signaling (M5923), p53 pathway (M5939), Peroxisome (M5949), Oxidative Phosphorylation (M5936), and Glycolysis (M5937) (Fig. 3F).

### Gene ontology analysis revealed that STEE-induced transcriptomic changes were associated with a wide range of biological events in C2C12 myotubes and HepG2 hepatocytes

To further investigate the potential regulatory effects of STEE on C2C12 myotubes and HepG2 hepatocytes, we performed gene ontology (GO) analysis. The analysis statistically tests whether the proportion of GO terms for a list of specific DEGs is significantly higher than the proportion of the terms for the population (enrichment). This allows us to detect characteristic terms for a group of DEGs, thereby supporting the capture of biological phenomena. GO term is represented by three sub-ontologies: biological process (BP), cellular component (CC), and molecular function (MF).

**Fig. 4:**
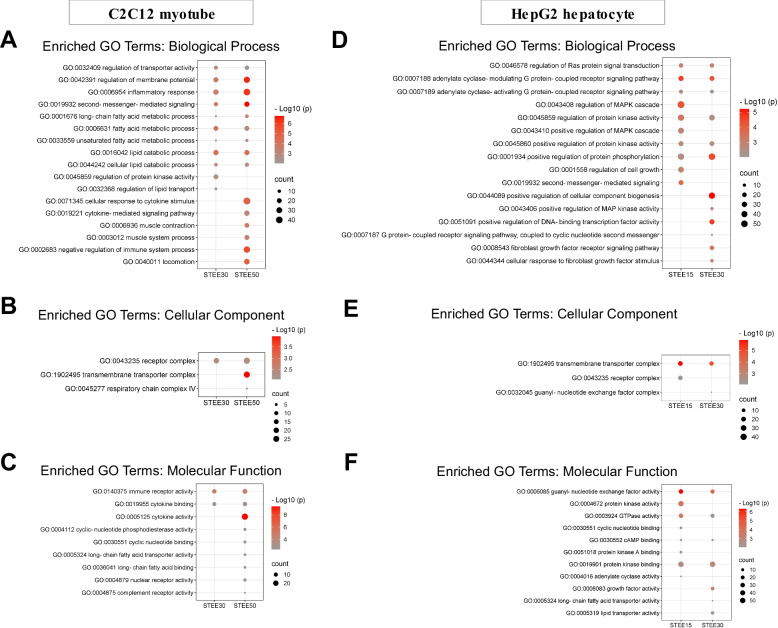
Enriched GO terms reflecting STEE-induced bio-phenomena in C2C12 and HepG2. GO analysis revealed enriched biological process (BP), cellular component (CC), and molecular function (MF) gene sets by the DEGs in STEE-treated C2C12 myotubes and HepG2 hepatocytes compared to their respective nontreated control groups. **A**-**C** Dot plots showing significantly enriched GO terms of BP, CC, and MF by the DEGs between STEE-treated C2C12 myotubes and control. **D**-**F** Dot plots showing significantly enriched GO terms of BP, CC, and MF by the DEGs between STEE-treated HepG2 cells and control. The size of the circle denotes the number of genes. The negative log10 of the *p*-value is represented by the color

As a result of GO analysis for the DEGs in STEE-treated C2C12 group, regulation of membrane potential (GO:0042391), inflammatory response (GO:0006954), second-messenger-mediated signaling (GO:0019932), long-chain fatty acid metabolic process (GO:0001676), fatty acid metabolic process (GO:0006631), and cellular lipid catabolic process (GO:0044242) were enriched as GOBP terms over-represented by the DEGs both in STEE30-M and STEE50-M (Fig. 4A). In addition, of the GOBPs, regulation of protein kinase activity (GO:0045859) and regulation of lipid transport (GO:0032368) were enriched by the DEGs in STEE30-M, and cellular response to cytokine stimulus (GO:0071345), cytokine-mediated signaling pathway (GO:0019221), muscle system process (GO:0003012), and negative regulation of immune system process (GO:0002683) were enriched by the DEGs in STEE50-M (Fig. 4A). Enrichment of GOBP terms for cytokine was unique to the DEGs in STEE50-M.

Of the GOCCs, receptor complex (GO:0043235) was enriched term over-represented by the DEGs both in STEE30-M and STEE50-M (Fig. 4B). Also, transmembrane transporter complex (GO:1902495) and respiratory chain complex IV (GO:0045277) were unique enriched terms over-represented by the DEGs in STEE50-M (Fig. 4B).

Of the GOMFs, immune receptor activity (GO:0140375) and cytokine binding (GO:0019955) were enriched terms over-represented by the DEGs both in STEE30-M and STEE50-M (Fig. 4C). Also, cytokine activity (GO:0005125), cyclic-nucleotide phosphodiesterase activity (GO:0004112), long-chain fatty acid transporter activity (GO:0005324), nuclear receptor activity (GO:0004879), and complement receptor activity (GO:0004875) were unique enriched terms over-represented by the DEGs in STEE50-M (Fig. 4C). Enrichment of GOMF terms for fatty acid, nuclear receptor, and complement was unique to the DEGs in STEE50-M.

A full list of enriched GO terms shown in Fig. 3A–C is given in Additional file 2: Table S1.

As a result of GO analysis for the DEGs in STEE-treated HepG2 group, regulation of Ras protein signal transduction (GO:0046578), adenylate cyclase-modulating G protein-coupled receptor signaling pathway (GO:0007188), regulation of protein kinase activity (GO:0045859), and positive regulation of protein kinase activity (GO:0045860) were enriched as GOBP terms over-represented by the DEGs both in STEE15-H and STEE30-H (Fig. 4D). In addition, of the GOBPs, regulation of MAPK cascade (GO:0043408), regulation of cell growth (GO:0001588), and second-messenger-mediated signalling (GO:0019932) were enriched by the DEGs in STEE15-H, and positive regulation of cellular component biogenesis (GO:0044089), positive regulation of MAP kinase activity (GO:043406), positive regulation of DNA-binding transcription factor activity (GO:0051091), and fibroblast growth factor receptor signaling pathway (GO:0008543) were enriched by the DEGs in STEE30-H (Fig. 4E). Enrichment of GOBP terms for fibroblast growth factor was unique to the DEGs in STEE30-H.

Of the GOCCs, transmembrane transporter complex (GO:1902495) was enriched term over-represented by the DEGs both in STEE30-M and STEE50-M (Fig. 4E). Also, receptor complex (GO:0043235) was unique enriched term over-represented by the DEGs in STEE15-H, and guanyl-nucleotide exchange factor complex (GO:0032045) was unique enriched term over-represented by the DEGs in STEE30-H (Fig. 4E).

Of the GOMFs, guanyl-nucleotide exchange factor activity (GO:0005085), GTPase activity (GO:0003924), cAMP binding (GO:0030552), and protein kinase binding (GO:0019910) were enriched terms over-represented by the DEGs both in STEE15-H and STEE30-H (Fig. 4F). Also, cyclic nucleotide binding (GO:0030551), protein kinase A binding (GO:0051018), and adenylate cyclase activity (GO:0004016) were enriched by the DEGs in STEE15-H, and growth factor activity (GO:0008083), long-chain fatty acid transporter activity (GO:0005324), and lipid transporter activity (GO:0005319) were enriched by the DEGs in STEE30-H (Fig. 4F). Enrichment of GOMF terms for growth factor and lipid transport were unique to the DEGs in STEE30-H.

A full list of enriched GO terms shown in Fig. 3D–F is given in Additional file 2: Table S2.

### The dimensionality reduction approach revealed that STEE regulated biological pathways related to lipid metabolism, protein kinase signaling, and cytokine signaling

Next, we performed the dimensionality reduction technique to retain latent properties for the large number of DEGs. The pathways clustered from the BioPlanet_2019 gene set library were then visualized on two dimensions using Uniform Manifold Approximation and Projection (UMAP), facilitating the classification of DEG sets involved in the biological pathways.

**Fig. 5:**
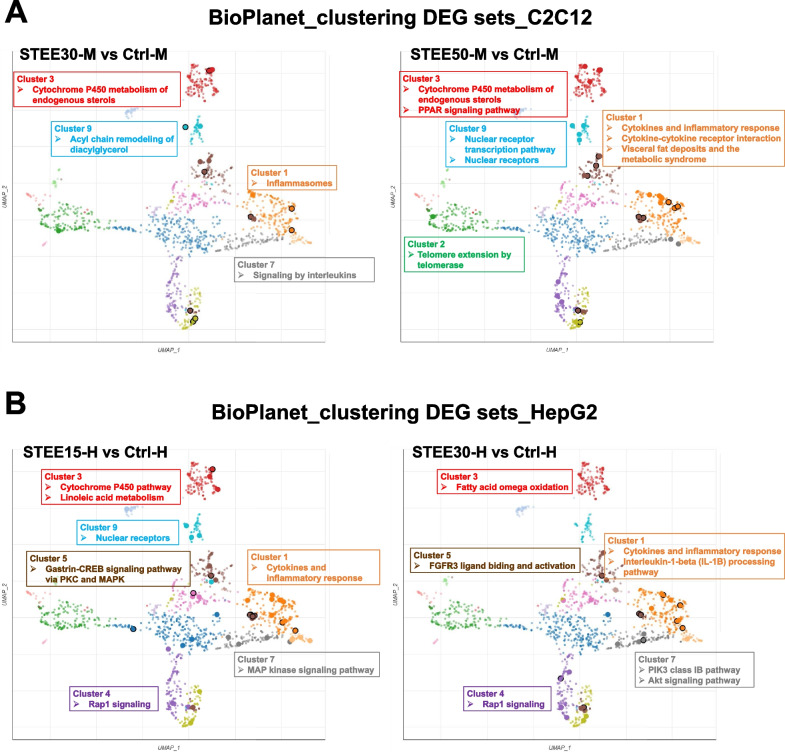
Pathways on the UMAP plots related to the transcriptomic modulation by STEE. Scatterplots showing similar pathway gene set clusters identified through the UMAP dimensionality reduction technique using the BioPlanet 2019 gene set library. **A** Pathway clusters enriched by the DEGs in STEE-treated C2C12 myotubes (both concentrations). **B** Pathway clusters enriched by the DEGs in STEE-treated C2C12 myotubes (both concentrations). The points (pathway terms) are gathered and color-coded by similarity or relevance. The size and the darkness of the circle denote the degree of enrichment

In the analysis for the DEGs in STEE-treated C2C12 groups, Inflammasomes (Cluster 1, orange), Cytochrome P450 metabolism of endogenous sterols (Cluster 3, red), Signaling by interleukins (Cluster 7, gray), and Acyl chain remodeling of diacylglycerol (Cluster 9, light blue) were detected as the related pathways over-represented by the sets of DEGs in STEE30-M (Fig. 5A). Also, Cytokines and inflammatory response (Cluster 1), Cytokine-cytokine receptor interaction (Cluster 1), Visceral fat deposits and the metabolic syndrome (Cluster 1), Telomere extension by telomerase (Cluster 2, green), Cytochrome P450 metabolism of endogenous sterols, PPAR signaling pathway (Cluster 3), Nuclear receptor transcription pathway (Cluster 9), and Nuclear receptors (Cluster 9) were detected as the related pathways over-represented by the sets of DEGs in STEE50-M (Fig. 5A). Cumulatively, these results suggested STEE may impact pathways related to cytokines, lipid metabolism, and nuclear receptors in C2C12 myotubes.

In the analysis for the DEGs in STEE-treated HepG2 groups, Cytokine and inflammatory response, Cytochrome P450 metabolism of endogenous sterols, Linoleic acid metabolism (Cluster 3), Rap1 signaling (Cluster 4, purple), Gastrin-CREB signaling pathway via PKC and MAPK (Cluster 5, brown), MAP kinase signaling pathway (Cluster 7), and Nuclear receptors were detected as the related pathways over-represented by the sets of DEGs in STEE15-H (Fig. 5B). Also, Cytokines and inflammatory response, Interleukin-1-beta (IL-1B) processing pathway (Cluster 1), fatty acid omega oxidation (Cluster 3), Rap signaling (Cluster 4), FGFR3 ligand binding and activation (Cluster 5), PIK3 class IB pathway (Cluster 7), and Akt signaling pathway (Cluster 7) were detected as the related pathways over-represented by the sets of DEGs in STEE30-H (Fig. 5B). Cumulatively, these results suggested STEE may impact pathways related to cytokines, lipid metabolism, and protein kinase signaling (especially MAP kinase) in HepG2 hepatocytes.

### Protein–protein interaction (PPI) networking of DEGs regulated by STEE

**Fig. 6:**
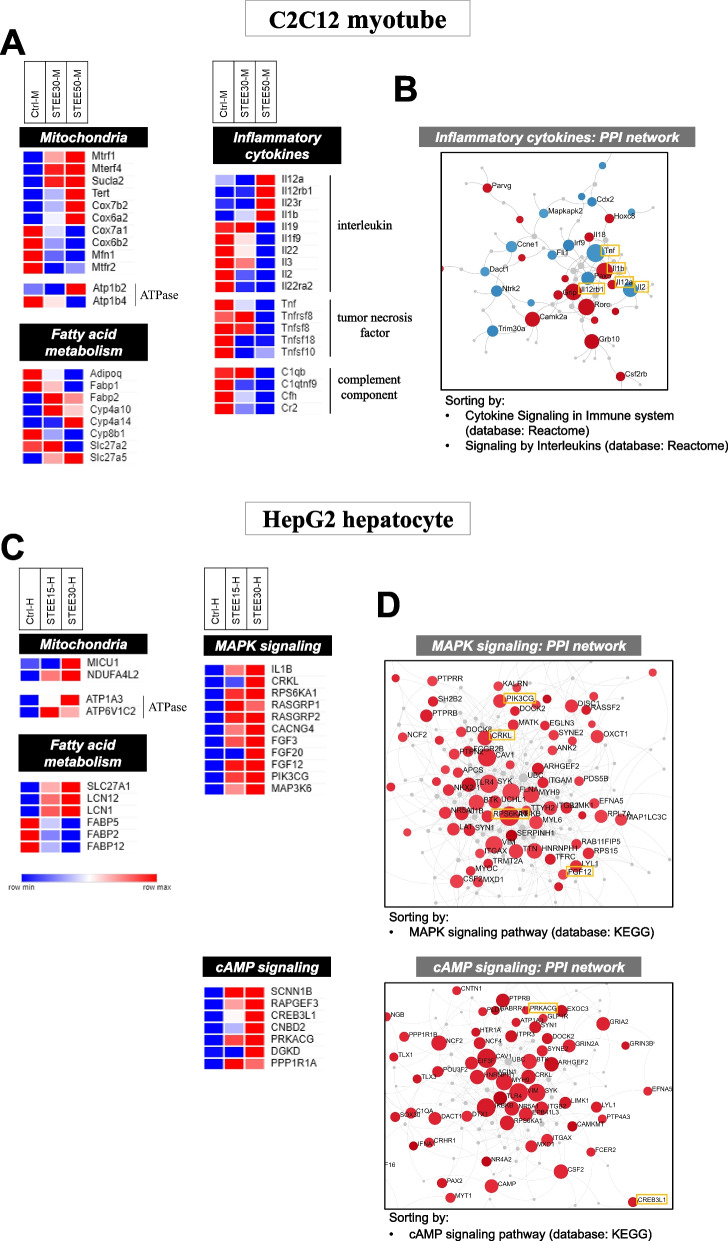
Differential expression heatmap and interaction of the genes. **A** Heatmaps comparing the mean expression of transcripts in STEE-treated (lower and higher concentrations) C2C12 and non-treated control. **B** A module from PPI networks of DEGs of inflammatory cytokines in STEE50-M. The red node denotes up-regulated DEGs and the blue node denotes down-regulated DEGs. **C** Heatmaps comparing the mean expression of transcripts in STEE-treated (lower and higher concentrations) HepG2 and non-treated control. **D** Modules from PPI networks of upregulated genes related MAPK signaling and cAMP signaling in STEE30-H. The red node denotes up-regulated genes. The bar represents color-toned relative expression levels. Color shading of the nodes indicates the relative intensity. The gray nodes represent the proteins

Given the indication of the analyses suggesting the modulation of biological events such as mitochondria activity, fatty acid (FA) metabolism, inflammatory response, and signal cascade of MAP kinase or cAMP by STEE, we attempted to do further investigation of each DEG and classify them based on their functions by using MSigDB and GeneCards. We chose to look at genes that were differentially expressed (satisfied with thresholds) in the samples treated with higher concentrations of the extract (STEE50-M and STEE30-H) compared to the controls. The heatmaps show the relative intensity of the genes (average of duplicates) regulated in C2C12 groups (Fig. 6A) and HepG2 groups (Fig. 6C).

We found that some mitochondria activity-related transcripts were differentially regulated by the STEE treatment. Among the genes related to mitochondrial respiration, *Cox7b2* and *Cox6a2* were significantly upregulated in STEE50-M, and *Cox7a1* and *Cox6b2* were significantly downregulated in STEE50-M. Respiratory electron transport-related *NDUFA4L2* showed significant upregulation in STEE30-H and showed 1.33-fold upregulation satisfied with *p* < 0.05 in STEE15-H. Two mitochondrial transcription and translation-related genes: *Mtrf1* and *Mterf4*, TCA cycle-related gene: *Sucla2*, and telomerase: *Tert* were significantly upregulated in STEE50-M. *Mterf4* was significantly downregulated also in STEE30-M. Of the two fusion and fission-related genes: *Mfn1* and *Mtfr2*, *Mtfr2* was significantly downregulated both in STEE30-M and STEE50-M, and *Mfn* showed significant downregulation in STEE50-M and also showed 1.24-fold downregulation tendency in STEE30-M (*p* = 0.0514). Furthermore, *Atp1b2*, an ion-transporting ATPase that plays a role in ATP generation by oxidative phosphorylation (OxPhos) [24–26], was significantly upregulated in STEE50-M. Another ATPase gene *Atp1b4* was significantly downregulated in STEE50-M. In STEE30-H, ion-transporting ATPase genes *ATP1A3* and *ATP6V1C2* were significantly upregulated. These two genes showed 1.38- and 1.42-fold upregulation satisfied with *p* < 0.05 in STEE15-H, respectively.

We found that STEE treatment resulted in a differential expression of some FA metabolism-related genes. The adipogenic differentiation-related *Adipoq* was significantly downregulated in STEE50-M. Among the FA transport-related genes, *Fabp1* and *Slc27a2* were significantly downregulated in STEE50-M, and *Fabp2* and *Slc27a5* were significantly upregulated in STEE50-M. *Fabp2* was also significantly upregulated in STEE30-M. The FA transporter genes: *FABP5*, *FABP2*, and *FABP12* were significantly downregulated in STEE30-H. Among those three genes, *FABP2* and *FABP12* showed 1.48-fold downregulation satisfied with *p* < 0.05 in STEE15-H, and *FABP5* showed 1.26-fold downregulation tendency in STEE15-H (*p* = 0.0677). Other FA transport-related genes: *SLC27A1*, *LCN12*, and *LCN1* were significantly upregulated in STEE-30-H. Among them, *SLC12A1* and *LCN1* showed 1.29- and 1.43-fold upregulation satisfied with *p* > 0.05 in STEE15-H, respectively. In addition, enzymatic metabolism-related genes: *Cyp4a10* and *Cyp4a14* were significantly upregulated and *Cyp8b1* was significantly downregulated in STEE50-M. Among them, *Cyp4a10* showed 1.26-fold upregulation tendency (*p* = 0.0575) and *Cyp8b1* showed 1.23-fold downregulation tendency (*p* = 0.0824) in STEE30-M, respectively.

We found some DEGs of inflammatory cytokines and their receptors from the data. Interleukin genes: *Il12a*, *Il12rb1*, *Il23r*, and *Il1b* showed significant upregulation, and *Il19*, *Il1f9*, *Il22*, *Il3*, *Il2*, and *Il22ra2* showed significant downregulation in STEE50-M. *Il22ra2* was significantly downregulated also in STEE30-M. Tumor necrosis factor genes: *Tnf*, *Tnfrsf18*, *Tnfsf8*, *Tnfsf18*, and *Tnfsf10* were significantly downregulated in STEE50-M. Among them, *Tnfsf18* showed 1.22-fold downregulation tendency (*p* = 0.0689) in STEE30-M. Complement component genes: *C1qb*, *C1qtnf9*, *Cfh*, and *Cr2* were significantly downregulated in STEE50-M. Among them, *C1qtnf9* showed 1.23-fold downregulation tendency (*p* = 0.0817) in STEE30-M. Many cytokine genes were significantly regulated in STEE50-M compared to STEE30-M suggesting a higher concentration of STEE may have a stronger impact on inflammatory modulation in C2C12 myotubes.

Given this result, next, we attempted to construct protein–protein interaction (PPI) networks to analyze the interactions between the DEGs. Initial sub-networks consisting of nodes (represent genes) and edges (represent interactions) were constructed based on the first-order generic PPI in the comparison between STEE50-M and Ctrl-M. Then the networks were sorted by 'Cytokine Signaling in Immune system' and 'Signaling by Interleukins' of the Reactome database [27], and a module consisting of 17 nodes represented by downregulated genes and 17 nodes by upregulated genes could be extracted. *Tnf*, *Il2*, *Il12rb1*, and *Il1b* were detected as cytokine genes with many edges in the module, i.e., high interaction in the module (Fig. 6B).

Also, we found some DEGs encoded for the molecules that constitute signaling pathways from the data. MAPK signaling-related genes: *IL1B*, *CRKL*, *RPS6KA1*, *RASGRP1*, *RASGRP2*, *CACNG4*, *PIK3GC*, and *MAP3K6*, and fibroblast growth factor genes: *FGF3*, *FGF20*, and *FGF12* showed significant upregulation in STEE30-H. Among them, *RASGRP1*, *RASGRP2*, *CACNG4*, *FGF3*, and *PIK3GC* were significantly upregulated also in STEE15-H. In addition, *IL1B*, *RPS6KA1*, *FGF12*, and *MAP3K6* showed 1.41-, 1.49-, 1,37-, and 1.22-fold upregulation satisfied with *p* < 0.05 in STEE15-H, respectively. cAMP signaling-related genes: *SCNN1B*, *RAPGEF3*, *CREB3L1*, *CNBD2*, *PRKACG*, *DGKD,* and *PPP1R1A* showed significant upregulation in STEE30-H. Among them, *SCNN1B*, *RAPGEF3*, *CNBD2*, and *PPP1R1A* showed significant upregulation also in STEE15-H, and *CREB3L1* showed 1,28-fold upregulation satisfied with *p* > 0.05 in STEE15-H.

Given these results, sub-networks of the upregulated genes related to MAPK signaling in STEE30-H were constructed by minimum-order generic PPI and sorted by 'MAPK signaling pathway' of the KEGG database [28]. We could extract a module consisting of 63 nodes represented by upregulated genes. Of the genes shown in the heatmap, *RPS6KA1*, *CRKL*, *PIK3CG*, and *FGF12* were presented in the module. In addition to these, the module was made up of many genes and proteins (Fig. 6D). Also, sub-networks of the upregulated genes related to cAMP signaling in STEE30-H were constructed by minimum-order generic PPI and sorted by 'cAMP signaling pathway' of the KEGG database. We could extract a module consisting of 35 nodes represented by upregulated genes. Of the genes shown in the heatmap, *PRKACG* and *CREB3L1* were presented in the module. The module was made up of many genes and proteins, and this included nodes of MAPK-related genes such as *RPS6KA1* and *CRKL* (Fig. 6D). These analyses indicated that the activated signaling pathways are constituted under the interaction of many genes, and even under the interaction between different pathways.

The list of DEGs presented in the heatmap is given in Additional file 2: Tables S3 and S4, together with the characteristics of each transcript.

### PPI analysis and qPCR approach suggested that STEE-induced transcriptional regulation by PGC-1α

Given the microarray data analysis indicating transcriptional regulation of nuclear receptor-related pathways (especially peroxisome proliferator-activated receptor gamma; PPARγ) by STEE, we attempted to construct PPI networks to analyze the interactions between the DEGs and the transcription factors (TFs), which could be regulated by STEE.

**Fig. 7:**
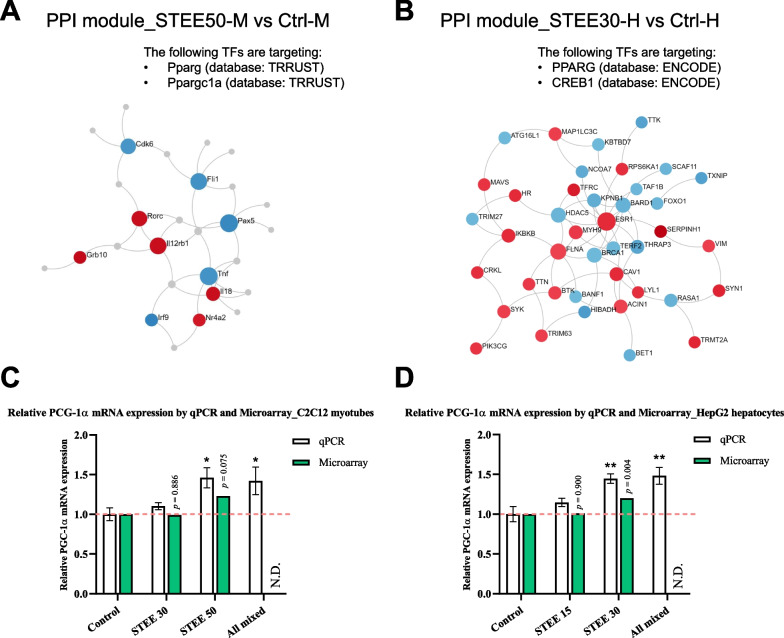
STEE regulates transcriptions linked with the transcription factor activity. **A** A module from PPI networks of DEGs between STEE50-M and Ctrl-M, which are targeted by TFs 'Pparg' and 'Ppargc1a' (TRRUST database). **B** A module from PPI networks of DEGs between STEE30-H and Ctrl-H, which are targeted by TFs 'PPARG' and 'CREB1' (ENCODE database). The red node denotes up-regulated DEGs and the blue node denotes down-regulated DEGs. Color shading of the nodes indicates the relative intensity. The gray nodes represent the proteins. **C**, **D** PGC-1α mRNA levels are expressed as relative values in STEE- or a mixture of STEE's polyphenols-treated C2C12 myotubes compared to control and in STEE- or a mixture of polyphenols-treated HepG2 cells compared to control. Values assessed by qPCR are shown in white (n = 4, except for two outliers in HepG2), and relative values assessed by the microarray are shown in green (n = 2). For qPCR data, error bars depict mean ± SEM, and One-way ANOVA with Dunnett's post hoc test was performed to assess statistical significance: *p < 0.05, **p < 0.01. N.D. means no data

To detect DEGs-TFs interactions in the comparison between STEE50-M and Ctrl-M, initial sub-networks were constructed based on the first-order generic PPI, and then the network regulated by the specific TFs was extracted as the module using the TRRSUT database [29]. We could extract a module consisting of 28 nodes having regulatory interaction with TFs 'Pparg' and 'Ppargc1a' (Fig. 7A). Of these nodes, five nodes were represented by downregulated genes, including *Il12rb1*, and five nodes by upregulated genes, including *Tnf* (other 18 nodes are proteins that had a high interaction with the up and downregulated DEGs).

Next, to detect DEGs-TFs interactions in the comparison between STEE30-H and Ctrl-H, initial sub-networks were constructed based on the zero-order generic PPI, and then the network regulated by the specific TFs was extracted as the module using the ENCODE database [30]. We could extract a module consisting of 19 nodes represented by downregulated genes and 22 nodes represented by upregulated genes, which have regulatory interaction with TFs 'PPARG' and 'CREB1' (Fig. 7B). These nodes included *ESR1* (up) and *FOXO1* (down), which have a tight relationship with PGC-1α [31–33]. Also, upregulated *RPS6KA1*, *CRKL*, and *PIK3CG* associated with the MAPK cascade were included in the module's nodes.

Microarray data analysis suggested that STEE has some effect on the activity of TFs, particularly those involved in PPARγ. We checked the dataset by focusing on PPARγ-related TFs and found that *ppargc1a* levels showed a 1.23-fold increase (*p* = 0.075) in STEE50-M compared with control (Fig. 7C), and *PPARGC1A* levels showed a 1.2-fold increase (*p* = 0.004) in STEE30-H compared with control (Fig. 7D), although outside the thresholds setting in the analysis. We, therefore, attempted to analyze the PGC-1α transcript levels by running PCR cycles with an increased number of biological replicates of each group.

In qPCR, additional two replicates were added to the two replicates used for the microarrays (*n* = 4 replicates for each). In addition, RNA samples from each cell treated with the STEE's four polyphenol mixture (All mixed) were added to this analysis: for the treatment to C2C12, 50 µg/mL STEE equivalents were treated for 6 h, and for the treatment to HepG2, 30 µg/mL STEE equivalents were treated for 24 h. As a result, the groups treated with the higher concentration-STEE (50 µg/mL in C2C12; 30 µg/mL in HepG2) and All mixed showed a statistically significant increase of *PGC-1α* expression both in C2C12 (approximately 1.4-fold; *p* > 0.05, respectively, Fig. 7C) and in HepG2 (approximately 1.45-fold; *p* > 0.01, respectively, Fig. 7D) compared to controls, indicating that running the PCR cycle with four biological replicates let us confirm a significant up-regulation of *PGC-1α* not only by STEE but also by its polyphenols.

Cumulatively, these results indicated STEE and its polyphenols may induce the physiological activations related to transcriptional activation of PGC-1α.

## Discussion

In the present study, we have demonstrated that STEE and its polyphenols could enhance mitochondrial activity in cultured myotubes and hepatocytes in vitro. Further, microarray-based omics analysis provides compelling evidence indicating that STEE could modulate an array of biological processes, physiological responses, and molecular pathways. Additionally, qPCR data validated that STEE and its polyphenols have the potential to bolster the activity of the pivotal mitochondrial master regulator, PGC-1α, within an in vitro context.

**Fig. 8:**
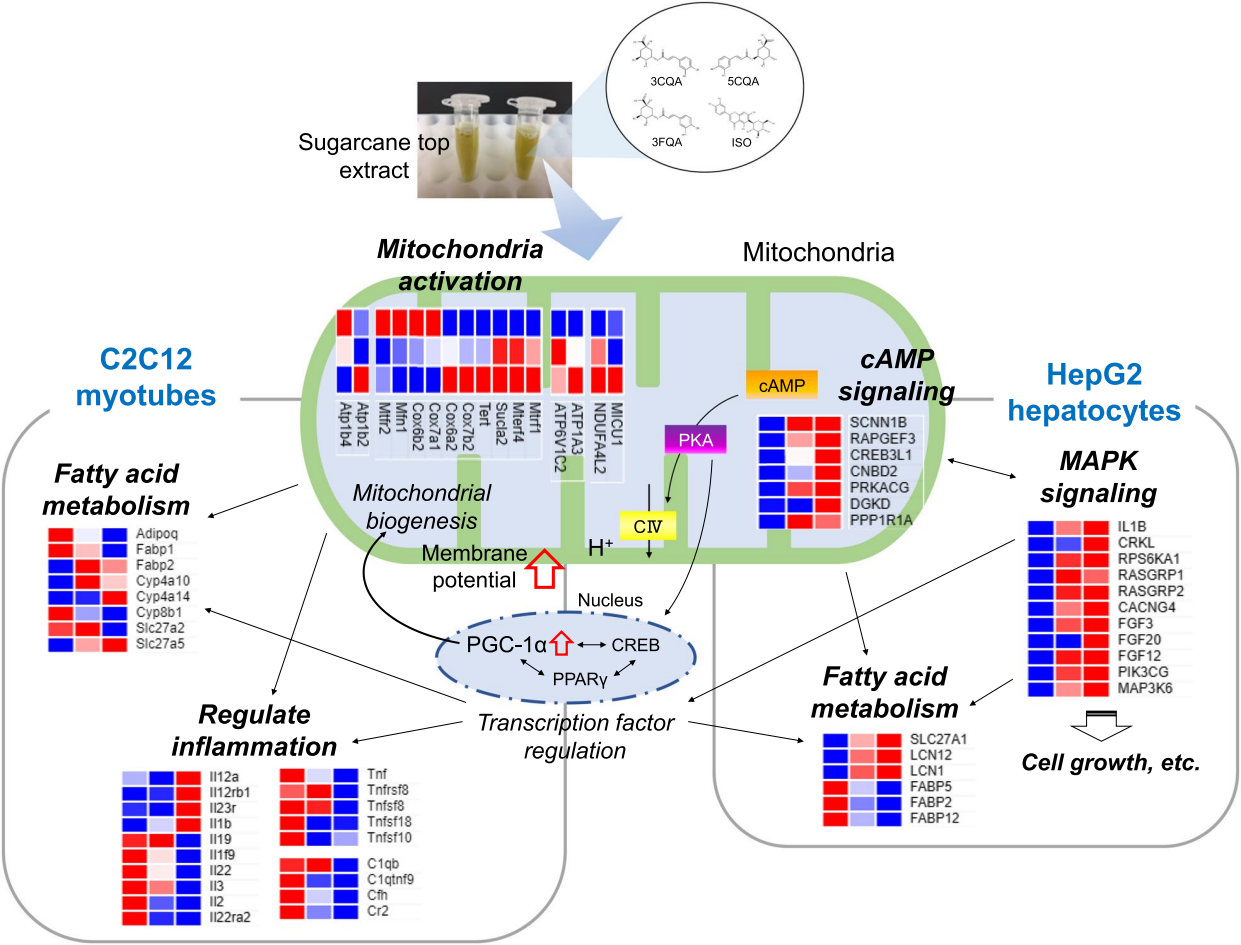
The predicted diagram of mitochondria and its related pathway modulation by STEE and its polyphenols. STEE and its polyphenols may stimulate the mitochondria activity, cAMP pathway, or transcription factor activity, especially PGC-1α. This activation could trigger other pathways activation such as fatty acid metabolism, inflammatory responses, and MAPK signaling

In a previous study, we documented the ability of STEE to facilitate astrocyte morphogenesis [14]. Notably, the mitochondrial activity, the cAMP pathway, and PGC-1α, which have been indicated as potential targets activated by STEE in the current research, have also been recognized as significant elements in the mechanisms underlying astrocyte stellation [34–36]. This suggests that these factors likely assume a central role in the regulatory effects elicited by STEE and its polyphenolic constituents. The findings of this study are summarized in Fig. 8.

The present study suggests a preparative action of second messenger-mediating cascades by STEE, particularly in HepG2 cells: adenylyl cyclase (AC) activity, AC-activating receptor signaling, guanyl-nucleotide exchange factor activity, and cyclic nucleotide signaling as second messenger were characteristics of bio-phenomena enriched by the DEGs in STEE-treated HepG2 cells. cAMP is a ubiquitous second messenger and is generated from ATP via the action of the AC [37]. cAMP activates its downstream effectors such as protein kinase A (PKA) or exchange protein activated by cAMP (EPAC) [38]. Upregulation of ion-transporting ATPase genes *ATP1A3* and *ATP6V1C2* and cAMP downstream effector-related genes *PRKACG*, *RAPGEF3* (EPAC encoding), and *PPP1R1A* (Inhibitor-1; I-1 encoding [39]) strongly suggest that STEE exposure activated ATP generation and following cAMP pathway in HepG2 cells. Also, PKA in the matrix induces phosphorylation of mitochondrial substrates including complex IV (cytochrome c oxidase; COX) [40–42]. Upregulation of ubiquinone subcomplex gene *NDUFA4L2* indicate that STEE may have a regulatory effect on the mitochondrial electron transport chain by modulating cAMP signaling [40, 42–44]. The previously reported data of soluble AC can be localized in the mitochondrial matrix reinforce the hypothesis of activation of the intramitochondrial second messenger cascade by STEE [44].

The TFs associated with nuclear receptors were suggested to be promising elements related to STEE's regulatory effects. Our findings confirmed the increase of PGC-1α expression by STEE. PGC-1α is a transcriptional coactivator that interacts with PPARγ, but also is the master regulator of mitochondrial biogenesis and plays a key role in metabolic homeostasis [5–7]. In energy metabolism, PGC-1α and its downstream effectors activate the mitochondrial complexes and OxPhos [45, 46]. The PPI analysis in this study gave a module targeted by the cAMP response element-binding protein (CREB), which is one of several PGC-1α upstream regulators. Also, CREB can be a downstream effector of PKA [9, 47]. Based on these backgrounds and the present data, STEE and its polyphenols could contribute to the cAMP-mediated activation of PGC-1α. Also, increased OxPhos activity could reduce mitochondrial reactive oxygen species (ROS) generation [48, 49], and mitochondrial ROS levels could affect its fusion and fission dynamics [50]. The balance between mitochondrial fusion and fission is critical to its quality control [2, 4]. Data in this study showed decreased levels of fusion- and fission-related genes *Mfn1* and *Mtfr2* [51], suggesting STEE may contribute more to activating mitochondrial biogenesis compared with mitochondrial dynamics. Furthermore, decreased PGC-1α is linked to cellular senescence with telomere shortening and DNA damage, and upregulation of telomerase reserve transcriptase (TERT)-encoding *Tret* suggests that STEE and its polyphenols may have a PGC-1α-mediated anti-cellular senescence effect [52, 53].

The transcriptomic analysis of this study suggested that FA metabolism was also targeted by STEE's bioactivity. FAs are generally a mitochondrial energy source. Intracellular FAs are converted into fatty acyl-CoA by acyl-CoA synthetase-activity of fatty acid transport proteins (FATPs), which are a family of trans-membrane transport proteins [54, 55]. Fatty acyl-CoA passed through the mitochondrial outer membrane and transported to the matrix is converted to acetyl-CoA by β-oxidation with enzymatic reactions. Excessive cytoplasmic FAs due to imbalances in energy demand increase oxidative stress and disrupt mitochondrial respiration [56, 57]. Upregulated FATP-encoding *SLC27A* genes suggest active FA transport in STEE-treated HepG2 cells [58]. However, data in this study showed that downregulated *FABP* genes, which is another FA transport protein [55, 59] in HepG2 cells, and upregulated microsomal ω-oxidation cytochrome P450 *Cyp4a* genes, which can generate ROS by its catalytic cycle [60, 61], in STEE-treated C2C12 myotubes. These suggest that STEE may affect fatty acid metabolism via minor pathways.

An environment of permanent oxidative stress could induce chronic inflammatory states [62]. Inflammation is the protective response to biological stimuli, and the signaling of cytokine, a small soluble peptide, fundamentally affects the induction and progression of inflammation. Transcription of cytokines is stimulated by cellular pathways including c-Jun N-terminal kinase (JNK) and p38 MAPK, which could be activated by oxidative stress [63]. Skeletal muscles are the primary site affected by age-related inflammation, and contractile dysfunction due to TNFα, a major endocrine stimulus, and imbalanced ROS production causes the decrease of muscle mass, strength, and quality [64–66]. Also, complements recruited by the immune system have been reported to play a key role in the pathogenesis of autoimmune muscle disorders such as inflammatory myopathies [67, 68]. The classical pathway components including C1 and C2 have been reported to be biosynthesized in myoblast cell lines [69]. Our microarray data suggest that STEE could act in an inhibitory manner on these pathways in in vitro myotubes.

Interleukins (e.g., IL-6) are also key cytokines that mediate chronic inflammation and subsequent muscle atrophy [66]. Of the microarray data of myotubes, *Il2*, *Il3*, and *Il12* were detected as DEGs encoded for the class I cytokine receptor family-binding molecules, and *Il19* and *Il22* were detected as DEGs encoded for the class II cytokine receptor family-binding molecules. Anti-inflammatory interleukins have been suggested to be elevated compensatory to an increase in pro-inflammatory ones, indicating that not only pro-inflammatory but also anti-inflammatory interleukin expression may be involved in the progression of myositis [70]. Interestingly, expression of the pro-inflammatory cytokine *IL-1β* gene was significantly upregulated in both types of STEE-treated cells. The previous studies reported that p38 MAPK can be phosphorylated by IL-1R-mediated signaling and that PGC-1α stimulated by ROS shows an anti-oxidative stress effect through a negative feedback loop [71, 72]. In addition, previous studies demonstrated that increases in PGC-1α protein levels would occur in parallel with an increase in the p-p38/p38 ratio in C2C12 by the *Gynostemma pentaphyllum* plant extract and HepG2 by the *Rosa roxburghii* Tratt seed oil [73, 74]. These reports and our previous findings [14] suggest that STEE and its polyphenols may induce an increase in PGC-1α protein levels with an increase in the p-p38/p38 ratio. These encourage us to further explore of the cytokine's defense system alterations by STEE and its polyphenols and related signaling molecule status [14].

Although the results of the assay with Rh123 reported in this and our previous study suggested that 3CQA, 5CQA, and ISO may be responsible for mitochondrial stimulation [14], there are other studies reported that 3FQA showed a strong relation to antioxidant-related proteins [75, 76], suggesting 3FQA may contribute to anti-oxidative stress, not via direct stimulation of mitochondria or PGC-1α.

Several MAPK signaling-related terms were enriched by the DEGs, particularly in STEE-treated HepG2 cells. Among MAPK cascade-related DEGs, the *FGF* genes showed up-regulation by STEE. The FGF family comprises signaling that stimulates various biological processes such as growth, differentiation, inflammation, or cellular senescence. The formation of complex FGF and its receptor FGFR phosphorylates the specific intercellular receptor domain and recruits other proteins like CRKL, which activates down-stream pathways such as Ras/MAPK or phosphatidylinositol 3-kinase (PI3K)/Akt signaling [77, 78]. Microarray data in this study suggest that STEE could regulate FGFR-mediated pathways in HepG2 cells, resulting in biological events such as cell growth. Data also showed upregulation of not only canonical FGF (*FGF3* and *FGF20*) but also intracellular FGF (*FGF12*) transcript levels in STEE-treated cells, suggesting STEE could regulate the channel activity [79].

The current study presents an integrated evaluation of transcriptional changes induced by STEE in both myotubes and hepatocytes, encompassing two different concentrations for each cell type. The findings suggest subtle differences in the activated signaling pathways between the two cell types, possibly attributable to inherent disparities in the nature of these cell lines originating from distinct animal species. For example, the previous study comparing gene expression profiles of mouse and human embryonic stem cells has suggested that differences in cytokine expression between human and mouse stem cells were species-specific rather than differences in culture conditions [80]. Also, a prior study examining the varying susceptibility of statins, commonly employed for cardiovascular disease prevention, in C2C12 and HepG2 cells revealed distinctive responses. Specifically, statins reduced phosphorylation of Akt (protein kinase B) and mitochondrial respiration in C2C12 myotubes but did not impact Akt signaling in HepG2 cells [81]. Nonetheless, noteworthy changes induced by STEE were observed in both cell lines, with C2C12 displaying alterations in genes related to cAMP and MAPK, and HepG2 exhibiting changes in cytokine genes. Inflammation and molecular pathways such as cAMP and MAPK could be mutually regulated rather than independently, as indicated by the PPI analyses in this study. This suggests that regulatory mechanisms could be established through interactions among factors, including the genes falling outside the specified cut-off values.

Nevertheless, the data predominantly pertain to transcript-level observations, prompting the need for evaluations at the protein or functional levels employing analytical techniques like flux analyzers. Furthermore, there is potential for an expanded inquiry aimed at elucidating with precision which specific compounds, including any synergistic effects arising from their combinations, target particular pathways and molecular mechanisms. It would also be valuable to validate the observed bioactivities within the context of stress conditions, such as oxidative stress. Finally, predicting the bioavailability and metabolism of polyphenols and their practical application in vivo remains challenging. Nonetheless, it is worth mentioning that prior clinical studies have documented the presence of unchanged CQAs and ISO in plasma following oral administration [82, 83].

## Conclusion

This study reported for the first time the regulation of mitochondrial activity in C2C12 myotubes and HepG2 hepatocytes following exposure to STEE and its polyphenols. An in-depth analysis of the microarray data has shed light on the multifaceted alterations in gene expression induced by STEE, implicating a wide array of biological processes such as mitochondrial function, FA metabolism, inflammatory cytokine responses, MAPK signaling, and cAMP signaling. Our findings further confirmed that STEE and its polyphenols stimulate the transcription of PGC-1α, a master regulator with pivotal roles in mitochondria. Taken together, these findings introduce STEE as a compelling candidate capable of imparting beneficial effects on both muscle and liver tissues. As we embark on future investigations, the impact of STEE and its polyphenols on muscle and liver functions in animal models will be further elucidated, potentially paving the way for their clinical applications.

## Materials and methods

### Preparation of STEE and chemical reagents

STEE and pure polyphenolic compounds were prepared as previously reported [14]. The freeze-dried amorphous powder was re-suspended in 70% (*v/v*) ethanol at 100 mg/mL. Pure STEE polyphenols 3CQA (≧98%), 5CQA (≧99%), 3FQA (≧98%), and ISO (≧98%) were purchased from Nagara Science (Gifu, Japan) or Sigma-Aldrich (St. Louis, MO, USA) and suspended in 70% (*v/v*) ethanol at 25 or 50 mM. The samples were divided into small aliquots and stored at − 80 °C.

### Cells and cell culture

C2C12 mouse myoblasts and HepG2 human hepatocytes were obtained from American Type Culture Collection (ATCC, Manassas, VA, USA).

C2C12 myoblasts were cultured at 37 °C under 5% CO_2_ in Dulbecco's modified Eagle's medium (DMEM) supplemented with 10% fetal bovine serum (FBS; Gibco-Thermo Fisher, Grand Island, NY, USA) and 1% anti-bacterial penicillin/streptomycin (PS). To induce differentiation, the growth medium was replaced with a differentiation medium composed of DMEM supplemented with 2% horse serum (Gibco) and 1% penicillin/streptomycin when cells reached about 90% confluence. After differentiation for 6 days, C2C12 myotubes were treated with samples and then subjected to the experiments.

HepG2 cells were maintained in DMEM containing 10% FBS and 1% PS under 5% CO_2_ at 37 °C. After the cells reached about 90% confluence, the cells were treated with samples and then subjected to the experiments.

### 3-(4,5-Dimethylthiazol-2-yl)-2,5-diphenyltetrazolium bromide (MTT) assay

The MTT assay was performed as a viability assay. Mitochondrial reductase converts the water-soluble yellow MTT to the insoluble purple formazan, and this allows one to detect cellular viability as changes in metabolic activity [84].

C2C12 myotubes or HepG2 cells cultured on the collagen-coated 96-well plate were treated with the extract at a range from 5 to 100 µg/mL for 24 or 48 h. The extract was diluted in serum-free Opti-MEM (Gibco) and used. After removing cultures, MTT solution (5 mg/mL) was added to each well for 3 h to let formazan crystals form, and then 10% SDS was added and incubated for 16 h in the dark to dissolve the crystals. The optical density (OD) was measured with a plate reader (Varioskan LUX, Thermo Fisher Scientific, Rockford, IL, USA) at 570 nm.

### Measurement of mitochondrial activity

Using Rh123, we evaluated intercellular mitochondrial activity. Rh123 is a green fluorescent dye monitoring the proton (H^+^) in mitochondrial intermembrane space, and fluorescence intensity from intercellular Rh123 (i.e., MMP) is proportionate to its mitochondrial activity [85, 86].

C2C12 myotubes or HepG2 cells cultured on the collagen-coated 96 wells plate were treated with the extract (50 µg/mL) or its bioactive compounds (equivalent to that contained in 50 µg/mL of the extract) for 6 or 24 h. The samples were diluted in serum-free Opti-MEM (Gibco) and used. After removing cultures, the cells were incubated with Rh123 solution (10 µg/mL) for 20 min at 37 °C. After washing with PBS, the cells lysed with 1% Triton-X solution for 30 min in the dark, and then transferred into a black clear-bottom 96-well plate. The fluorescence intensity was measured with a plate reader (Varioskan LUX, Thermo Fisher Scientific) at λex/λem = 507 nm/529 nm.

### RNA isolation

Total RNA was isolated from the cells using the RNeasy plus mini kit (Qiagen, Hilden, Germany) according to the manufacturer's instructions. Before experimentation, cells cultured on collagen-coated 6 well plates were treated with the extract or its compounds dissolved in serum-free Opti-MEM (Gibco) for 6 or 24 h. RNA concentration and quality were assessed by Nanodrop One (ThermoFisher Scientific).

### Microarray experiment

Microarray workflow was carried out using GeneChip™ WT PLUS Reagent Kit and GeneChip™ Hybridization, Wash and Stain Kit (Applied Biosystems-ThermoFisher, Foster City, CA, USA), with the protocol provided by the manufacturer. The starting material of 100 ng RNA was reverse transcribed to synthesize single strand-cDNA, and then the strands were fragmented and biotin-labeled. Fragmented, labeled strands were hybridized to probes on Mouse or Human Clariom S Assay chip for 16 h at 45 °C in GeneChip™ Hybridization Oven 645 (Affymetrix-ThermoFisher, Santa Clara, CA, USA). The hybridized chip was washed and stained on the GeneChip™ Fluidics Station 450, and then scanned on the GeneChip™ Scanner 3000.

### Microarray data analysis

Data processing was conducted using Transcriptome Analysis Console (TAC) version 4.0 and subjected to normalization employing the signal space transformation Robust Multiple Average (SST-RMA) package algorithm. DEGs were identified by comparing two mRNA biological samples within each group, employing a significance threshold of *p*-value < 0.05 (determined through one-way between-subjects ANOVA). For C2C12 myotubes, DEGs were defined based on a log2-fold change (FC) cutoff greater than 1.2 or smaller than − 1.2, while for HepG2 cells, DEGs were determined with a log2-FC cutoff exceeding 1.5 or falling below − 1.5.

GO terms over-represented by the DEGs were identified using the Metascape web tool (https://metascape.org/) [87]. The BioPlanet_2019 gene set library was used for clustering DEG by the biological pathways under the Enrichr online tool (https://maayanlab.cloud/Enrichr/) [88–90]. Term Frequency-Inverse Document Frequency (TF-IDF) values were calculated for each gene set, and the values were dimensionally reduced using the UMAP technique. The Leiden algorithm applied to the TF-IDF values identified the terms as a cluster, and the plotted clusters were assigned colors. The Molecular Signatures Database (MSigDB) of Gene Set Enrichment Analysis (GSEA) web tool (https://www.gsea-msigdb.org/gsea/index.jsp) and GeneCards database (https://www.genecards.org/) were used to annotate and analyze the functions of the DEGs. PPI sub-networks were built from the DEGs based on the IMEx Interactome database [91]. The modules were extracted from the network using the Transcription Explorer command, which detects regulatory interactions between the target factors and the target genes. These processes were done on the Network Analyst tool (https://www.networkanalyst.ca/NetworkAnalyst/home.xhtml) [92].

To generate heatmaps, we used Morpheus software (http://software.broadinstitute.org/Morpheus). Venn diagrams were generated using an open-source tool (http://bioinformatics.psb.ugent.be/webtools/Venn/). Butterfly bar charts, code diagrams, and dot plots were drawn using the bioinformatics online tool (https://www.bioinformatics.com.cn/).

Microarray data were deposited at Gene Expression Omnibus (GEO; accession no. GSE243411 for the C2C12 group dataset and GSE243412 for the HepG2 group dataset).

### Real-time quantitative polymerase chain reaction (RT-qPCR)

DNA synthesis was performed by using SuperScript IV VILO Master Mix (Applied Biosystems, Foster City, CA, USA) according to the manufacturer's protocol. The qPCR by the cycles of single-stranding (15 s at 95 °C), primer annealing (1 min at 60 °C), and amplification with Taq DNA polymerase (1 min at 72 °C) was run on Applied Biosystem's 7500 RT-PCR System. Used primers were as follows: *Ppargc1* (Mm01208835_m1), *Gapdh* (Mm99999915_g1), *PPARGC1* (Hs00173304_m1), *GAPDH* (Hs02786624_g1). We chose *Gapdh* or *GAPDH* as housekeeping control to normalize the cycle threshold (CT) values of the target transcript calculated by the ΔΔCT method.

### Statistical analysis

All statistical analyses were performed using GraphPad Prism 8 (GraphPad, San Diego, CA, USA). Data were tested for normality by the Shapiro–Wilk test. A one-way analysis of variance (ANOVA) followed by Dunnett's post hoc test was performed on normally distributed data to compare the experimental groups against a control group. The Kruskal−Wallis test followed by Dunn's post hoc test was performed on non-normally distributed data. The significance level was set at α < 0.05 in all cases. Error bars depict mean ± standard error of means (SEM).

### Supplementary Information

**Additional file 1: Fig. S1.** STEE treatment did not affect the viability of both C2C12 and HepG2. (A) C2C12 myotubes were treated with ranged concentrations of STEE (5, 15, 30, 50, 70, and 100 µg/mL) for 24 or 48 h, and then the OD of the cell lysate including MTT was determined as an indication of the cell viability. (B) HepG2 hepatocytes were treated with ranged concentrations of STEE for 24 or 48 h, and then the OD was determined. Results are expressed as relative percentages compared with the control (mean ± SEM, n = 4-6). One-way ANOVA with Dunnett’s post hoc test was performed to assess statistical significance.

**Additional file 2:** **Table S1.** A list of GO terms presented in the dot plot of Figure 3A-C. **Table S2.** A list of GO terms presented in the dot plot of Figure 3D-F. **Table S3.** A list of the DEGs presented in the heatmap of Figure 6A. **Table S4.** A list of the DEGs presented in the heatmap of Figure 6C.

## Data Availability

The data generated and/or analyzed for this study are included in this article and its supplementary information files. Microarray data were deposited at NCBI GEO under accession no. GSE243411 for the C2C12 group dataset and GSE243412 for the HepG2 group dataset.
