# Supplementary figures and images for: Modulation of mitochondrial activity by sugarcane (Saccharum officinarum L.) top extract and its bioactive polyphenols: a comprehensive transcriptomics analysis in C2C12 myotubes and HepG2 hepatocytes
Source: Nat Prod Bioprospect. 2024 Jan 5;14(1):2. doi: 10.1007/s13659-023-00423-x (PMC10766937; doi:10.1007/s13659-023-00423-x)

**A**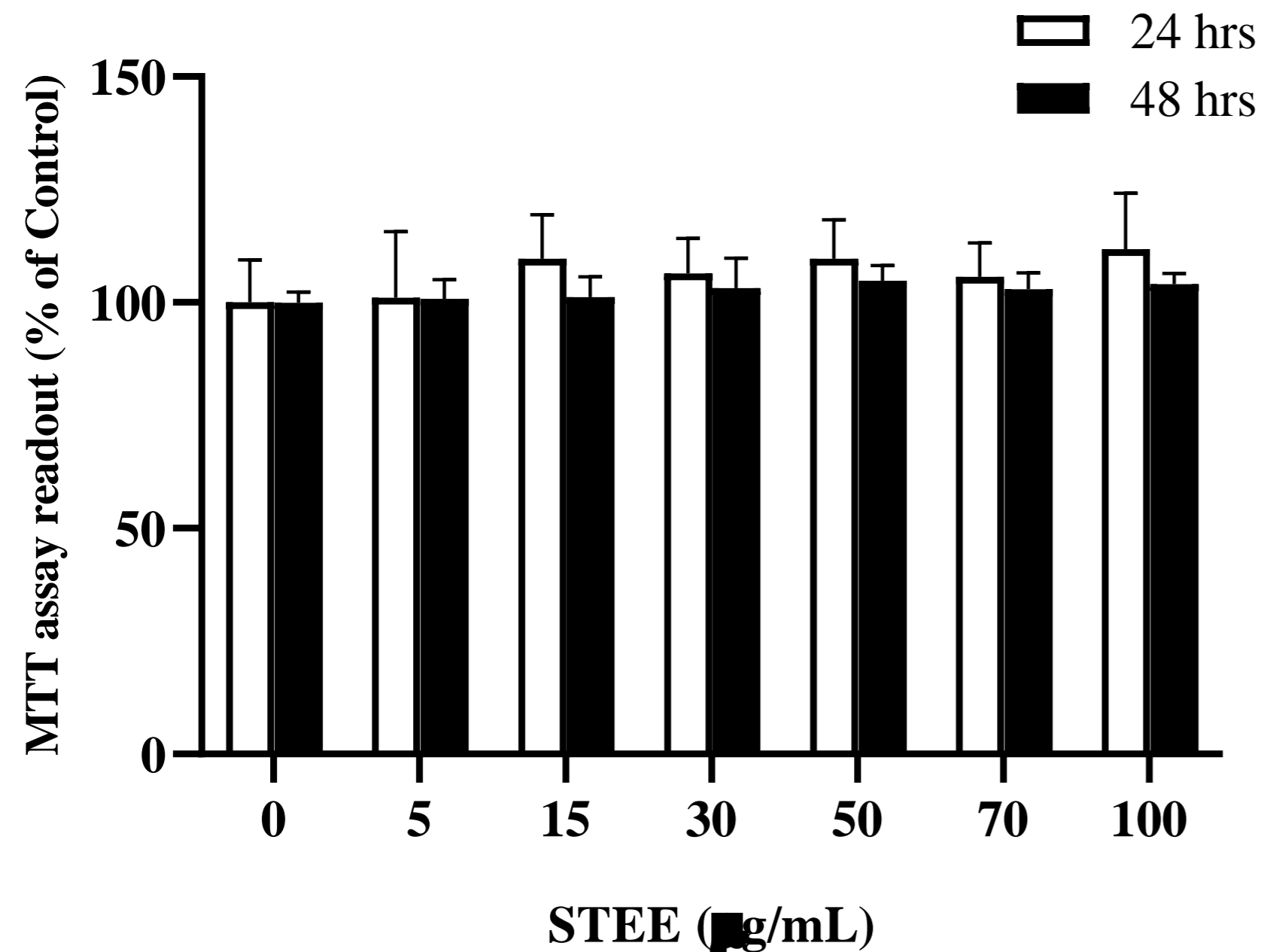**B**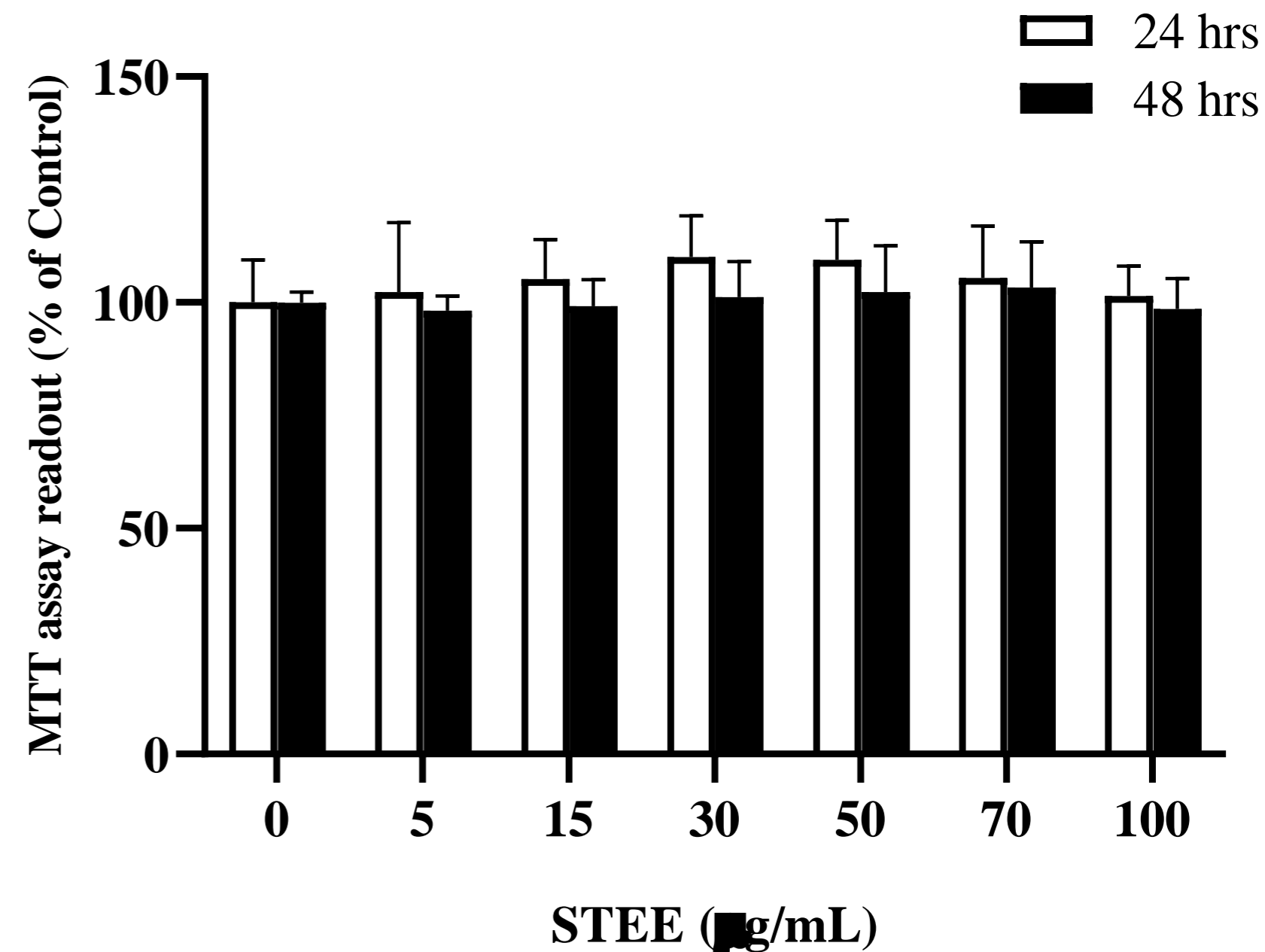

**Fig. S1.** STEE treatment did not affect the viability of both C2C12 and HepG2.

Supplement: Supplementary file 1 — Additional file 1: Fig. S1. STEE treatment did not affect the viability of both C2C12 and HepG2. (A) C2C12 myotubes were treated with ranged concentrations of STEE (5, 15, 30, 50, 70, and 100 µg/mL) for 24 or 48 h, and then the OD of the cell lysate including MTT was determined as an indication of the cell viability. (B) HepG2 hepatocytes were treated with ranged concentrations of STEE for 24 or 48 h, and then the OD was determined. Results are expressed as relative percentages compared with the control (mean ± SEM, n = 4-6). One-way ANOVA with Dunnett’s post hoc test was performed to assess statistical significance. [file 13659_2023_423_MOESM1_ESM.pdf]
