# Supplementary material for: Modulation of mitochondrial activity by sugarcane (Saccharum officinarum L.) top extract and its bioactive polyphenols: a comprehensive transcriptomics analysis in C2C12 myotubes and HepG2 hepatocytes
Source: Nat Prod Bioprospect. 2024 Jan 5;14(1):2. doi: 10.1007/s13659-023-00423-x (PMC10766937; doi:10.1007/s13659-023-00423-x)
Supplement: Supplementary file 2 — Additional file 2: Table S1. A list of GO terms presented in the dot plot of Figure 3A-C. Table S2. A list of GO terms presented in the dot plot of Figure 3D-F. Table S3. A list of the DEGs presented in the heatmap of Figure 6A. Table S4. A list of the DEGs presented in the heatmap of Figure 6C. [file 13659_2023_423_MOESM2_ESM.docx]

**Supplementary Tables**

**Table S1. A list of GO terms presented in the dot plot of Figure 3A-C.**

| **GO ID** | **GO term name** | **-log10 (*p*-value)** | | **# overlapped genes** | |
| --- | --- | --- | --- | --- | --- |
|  |  | **STEE30 vs Control** | **STEE50 vs Control** | **STEE30 vs Control** | **STEE50 vs Control** |
| **biological process** | | | | | |
| GO:0032409 | regulation of transporter activity | 2.61 | 2.19 | 13 | 16 |
| GO:0042391 | regulation of membrane potential | 3.84 | 6.75 | 21 | 23 |
| GO:0006594 | inflammatory response | 3.65 | 6.28 | 24 | 39 |
| GO:0019932 | second-messenger-mediated signaling | 3.95 | 6.75 | 14 | 23 |
| GO:0001676 | long-chain fatty acid metabolic process | 2.26 | 3.41 | 7 | 11 |
| GO:0006631 | fatty acid metabolic process | 4.20 | 2.84 | 18 | 20 |
| GO:0033559 | unsaturated fatty acid metabolic process | 2.42 | 2.49 | 7 | 9 |
| GO:0016042 | lipid catabolic process | 4.29 | 4.32 | 23 | 22 |
| GO:0044242 | cellular lipid catabolic process | 2.87 | 2.77 | 11 | 14 |
| GO:0045859 | regulation of protein kinase activity | 2.36 |  | 19 |  |
| GO:0032368 | regulation of lipid transport | 2.01 |  | 8 |  |
| GO:0071345 | cellular response to cytokine stimulus |  | 4.85 |  | 43 |
| GO:0019221 | cytokine-mediated signaling pathway |  | 3.07 |  | 23 |
| GO:0006936 | muscle contraction |  | 3.80 |  | 18 |
| GO:0003012 | muscle system process |  | 3.46 |  | 20 |
| GO:0002683 | negative regulation of immune system process |  | 5.71 |  | 36 |
| GO:0040011 | locomotion |  | 5.00 |  | 27 |
| **cellular component** | | | | | |
| GO:0043235 | receptor complex | 2.38 | 2.30 | 20 | 27 |
| GO:1902495 | transmembrane transporter complex | 3.95 |  | 26 |  |
| GO:0045277 | respiratory chain complex IV |  | 2.07 |  | 4 |
| **molecular function** | | | | | |
| GO:0140375 | immune receptor activity | 4.53 | 4.55 | 12 | 15 |
| GO:0019955 | cytokine binding | 2.69 | 2.93 | 9 | 12 |
| GO:0005125 | cytokine activity |  | 9.36 |  | 28 |
| GO:0004112 | cyclic-nucleotide phosphodiesterase activity |  | 3.08 |  | 5 |
| GO:0030551 | cyclic nucleotide binding |  | 2.37 |  | 5 |
| GO:0005324 | long-chain fatty acid transporter activity |  | 2.89 |  | 4 |
| GO:0036041 | long-chain fatty acid binding |  | 2.89 |  | 4 |
| GO:0004879 | nuclear receptor activity |  | 2.40 |  | 6 |
| GO:0004875 | complement receptor activity |  | 2.35 |  | 3 |

**Table S2. A list of GO terms presented in the dot plot of Figure 3D-F**

| **GO ID** | **GO term name** | **-log10 (*p*-value)** | | **# overlapped genes** | |
| --- | --- | --- | --- | --- | --- |
|  |  | **STEE15 vs Control** | **STEE30 vs Control** | **STEE15 vs Control** | **STEE30 vs Control** |
| **biological process** | | | | | |
| GO:0046578 | regulation of Ras protein signal transduction | 2.85 | 3.06 | 19 | 18 |
| GO:0007188 | adenylate cyclase-modulating G protein-coupled receptor signaling pathway | 4.65 | 4.22 | 27 | 24 |
| GO:0007189 | adenylate cyclase-activating G protein-coupled receptor signaling pathway | 2.36 | 2.03 | 15 | 13 |
| GO:0043408 | regulation of MAPK cascade | 4.25 |  | 56 |  |
| GO:0045859 | regulation of protein kinase activity | 3.38 | 2.48 | 42 | 35 |
| GO:0043410 | positive regulation of MAPK cascade | 2.79 |  | 38 |  |
| GO:0045860 | positive regulation of protein kinase activity | 2.60 | 2.32 | 25 | 22 |
| GO:0001934 | positive regulation of protein phosphorylation | 2.51 | 4.45 | 45 | 48 |
| GO:0001558 | regulation of cell growth | 2.95 |  | 35 |  |
| GO:0019932 | second-messenger-mediated signaling | 3.71 |  | 24 |  |
| GO:0044089 | positive regulation of cellular component biogenesis |  | 5.21 |  | 44 |
| GO:0043406 | positive regulation of MAP kinase activity |  | 2.37 |  | 10 |
| GO:0051091 | positive regulation of DNA-binding transcription factor activity |  | 4.74 |  | 26 |
| GO:0007187 | G protein-coupled receptor signaling pathway, coupled to cyclic nucleotide second messenger |  | 2.03 |  | 7 |
| GO:0008543 | fibroblast growth factor receptor signaling pathway |  | 3.19 |  | 19 |
| GO:0044344 | cellular response to fibroblast growth factor stimulus |  | 2.93 |  | 11 |
| **cellular component** | | | | | |
| GO:1902495 | transmembrane transporter complex | 5.85 | 4.78 | 42 | 36 |
| GO:0043235 | receptor complex | 2.11 |  | 39 |  |
| GO:0032045 | guanyl-nucleotide exchange factor complex |  | 2.30 |  | 4 |
| **molecular function** | | | | | |
| GO:0005085 | guanyl-nucleotide exchange factor activity | 6.33 | 4.43 | 30 | 24 |
| GO:0004672 | protein kinase activity | 3.70 |  | 47 |  |
| GO:0003924 | GTPase activity | 3.42 | 2.05 | 31 | 24 |
| GO:0030551 | cyclic nucleotide binding | 2.77 |  | 7 |  |
| GO:0030552 | cAMP binding | 2.38 | 2.61 | 5 | 5 |
| GO:0051018 | protein kinase A binding | 2.49 |  | 8 |  |
| GO:0019901 | protein kinase binding | 2.64 | 3.05 | 51 | 48 |
| GO:0004016 | adenylate cyclase activity | 2.21 |  | 4 |  |
| GO:0008083 | growth factor activity |  | 3.24 |  | 17 |
| GO:0005324 | long-chain fatty acid transporter activity |  | 2.30 |  | 4 |
| GO:0005319 | lipid transporter activity |  | 2.15 |  | 15 |

**Table S3. A list of the DEGs presented in the heatmap of Figure 6A.**

| **Gene symbol** | **Gene name** | **Fold change** | | ***p* value** | |
| --- | --- | --- | --- | --- | --- |
|  |  | **STEE30 vs Control** | **STEE50 vs Control** | **STEE30 vs Control** | **STEE50 vs Control** |
| ***Mitochondria related genes*** | | | | | |
| *Mtrf1* | mitochondrial translational release factor 1 | 1.21 | 1.36 | 0.1051 | 0.033 |
| *Mterf4* | mitochondrial transcription termination factor 4 | 1.28 | 1.35 | 0.0364 | 0.032 |
| *Sucla2* | succinate-Coenzyme A ligase, ADP-forming, beta subunit | 1.21 | 1.29 | 0.1748 | 0.0241 |
| *Tert* | telomerase reverse transcriptase | 1.04 | 1.29 | 0.7107 | 0.0475 |
| *Cox7b2* | cytochrome c oxidase subunit VIIb2 | -1.02 | 1.26 | 0.8651 | 0.0372 |
| *Cox6a2* | cytochrome c oxidase subunit VIa polypeptide 2 | 1.09 | 1.27 | 0.4407 | 0.0402 |
| *Cox7a1* | cytochrome c oxidase subunit VIIa 1 | -1.03 | -1.24 | 0.7179 | 0.0448 |
| *Cox6b2* | cytochrome c oxidase subunit VIb polypeptide 2 | -1.29 | -1.47 | 0.2138 | 0.0051 |
| *Mfn1* | mitofusin 1 | -1.24 | -1.3 | 0.0514 | 0.0479 |
| *Mtfr2* | mitochondrial fission regulator 2 | -1.54 | -1.37 | 0.0117 | 0.0182 |
| *Atp1b2* | ATPase, Na+/K+ transporting, beta 2 polypeptide | -1.09 | 1.4 | 0.416 | 0.0319 |
| *Atp1b4* | ATPase, (Na+)/K+ transporting, beta 4 polypeptide | -1.05 | -1.39 | 0.7874 | 0.0454 |
| ***Fatty acid metabolism related genes*** | | | | | |
| *Adipoq* | adiponectin, C1Q and collagen domain containing | -1.14 | -1.34 | 0.3819 | 0.0387 |
| *Fabp1* | fatty acid binding protein 1, liver | -1.12 | -1.36 | 0.2999 | 0.0136 |
| *Fabp2* | fatty acid binding protein 2, intestinal | 1.71 | 1.32 | 0.0047 | 0.0436 |
| *Cyp4a10* | cytochrome P450, family 4, subfamily a, polypeptide 10 | 1.26 | 1.26 | 0.0575 | 0.038 |
| *Cyp4a14* | cytochrome P450, family 4, subfamily a, polypeptide 14 | -1 | 1.26 | 0.9851 | 0.038 |
| *Cyp8b1* | cytochrome P450, family 8, subfamily b, polypeptide 1 | -1.23 | -1.31 | 0.0824 | 0.0364 |
| *Slc27a2* | solute carrier family 27 (fatty acid transporter), member 2 | 1.01 | -1.29 | 0.9316 | 0.03 |
| *Slc27a5* | solute carrier family 27 (fatty acid transporter), member 5 | 1.05 | 1.33 | 0.7497 | 0.021 |
| ***Immune and inflammatory signal related genes*** | | | | | |
| **Interleukin** | | | | | |
| *Il12a* | interleukin 12a | -1.09 | 1.34 | 0.3808 | 0.044 |
| *Il12rb1* | interleukin 12 receptor, beta 1 | 1.1 | 1.39 | 0.7258 | 0.0118 |
| *Il23r* | interleukin 23 receptor | 1.24 | 1.34 | 0.2937 | 0.015 |
| *Il1b* | interleukin 1 beta | 1.21 | 1.28 | 0.1065 | 0.0403 |
| *Il19* | interleukin 19 | 1.06 | -1.28 | 0.6371 | 0.0306 |
| *Il1f9* | interleukin 1 family, member 9 | -1.08 | -1.3 | 0.4961 | 0.0456 |
| *Il22* | interleukin 22 | -1.15 | -1.38 | 0.159 | 0.013 |
| *Il3* | interleukin 3 | -1.18 | -1.54 | 0.1548 | 0.0095 |
| *Il2* | interleukin 2 | 1.01 | -1.4 | 0.964 | 0.0367 |
| *Il22ra2* | interleukin 22 receptor, alpha 2 | -1.42 | -1.55 | 0.0091 | 0.0045 |
| **Tumor necrosis factor** | | | | | |
| *Tnf* | tumor necrosis factor | -1.08 | -1.32 | 0.625 | 0.0207 |
| *Tnfrsf8* | tumor necrosis factor receptor superfamily, member 8 | 1.03 | -1.33 | 0.7156 | 0.021 |
| *Tnfsf8* | tumor necrosis factor (ligand) superfamily, member 8 | 1.02 | -1.36 | 0.9304 | 0.0409 |
| *Tnfsf18* | tumor necrosis factor (ligand) superfamily, member 18 | -1.22 | -1.4 | 0.0689 | 0.0241 |
| *Tnfsf10* | tumor necrosis factor (ligand) superfamily, member 10 | -1.26 | -1.45 | 0.1622 | 0.0382 |
| **Complement component** | | | | | |
| *C1qb* | complement component 1, q subcomponent, beta polypeptide | 1.03 | -1.27 | 0.7592 | 0.0348 |
| *C1qtnf9* | C1q and tumor necrosis factor related protein 9 | -1.23 | -1.3 | 0.0817 | 0.0438 |
| *Cfh* | complement component factor h | -1.13 | -1.31 | 0.2287 | 0.0175 |
| *Cr2* | complement receptor 2 | 1.08 | -1.43 | 0.556 | 0.0056 |

**Table S4. A list of the DEGs presented in the heatmap of Figure 6C.**

| **Gene symbol** | **Gene name** | **Fold change** | | ***p* value** | |
| --- | --- | --- | --- | --- | --- |
|  |  | **STEE15 vs Control** | **STEE30 vs Control** | **STEE15 vs Control** | **STEE30 vs Control** |
| ***Mitochondria related genes*** | | | | | |
| *MICU1* | mitochondrial calcium uptake 1 | -1.13 | 1.66 | 0.1759 | 0.0081 |
| *NDUFA4L2* | NADH dehydrogenase (ubiquinone) 1 alpha subcomplex, 4-like 2 | 1.33 | 1.64 | 0.0064 | 0.0004 |
| *ATP1A3* | ATPase, Na+/K+ transporting, alpha 3 polypeptide | 1.38 | 1.69 | 0.0009 | 0.0004 |
| *ATP6V1C2* | ATPase, H+ transporting, lysosomal 42kDa, V1 subunit C2 | 1.42 | 1.66 | 0.0032 | 0.0067 |
| ***MAPK signal related genes*** | | | | | |
| *IL1B* | interleukin 1 beta | 1.41 | 1.79 | 0.0091 | 0.0004 |
| *CRKL* | CRK like proto-oncogene, adaptor protein | 1.11 | 1.71 | 0.0689 | 0.0002 |
| *RPS6KA1* | ribosomal protein S6 kinase, 90kDa, polypeptide 1 | 1.49 | 1.73 | 0.0014 | 0.0001 |
| *RASGRP1* | RAS guanyl releasing protein 1 (calcium and DAG-regulated) | 1.5 | 1.5 | 0.0316 | 0.0045 |
| *RASGRP2* | RAS guanyl releasing protein 2 (calcium and DAG-regulated) | 1.85 | 2.01 | 0.0007 | 3.01E-05 |
| *CACNG4* | calcium channel, voltage-dependent, gamma subunit 4 | 1.65 | 1.77 | 0.0006 | 0.0022 |
| *FGF3* | fibroblast growth factor 3 | 1.7 | 1.71 | 0.0002 | 0.0101 |
| *FGF20* | fibroblast growth factor 20 | 1.07 | 1.63 | 0.3579 | 0.0023 |
| *FGF12* | fibroblast growth factor 12 | 1.37 | 1.51 | 0.0085 | 0.0087 |
| *PIK3CG* | phosphatidylinositol-4,5-bisphosphate 3-kinase catalytic subunit gamma | 1.57 | 1.71 | 0.0214 | 0.0002 |
| *MAP3K6* | mitogen-activated protein kinase kinase kinase 6 | 1.22 | 1.54 | 0.0113 | 0.0002 |
| ***cAMP signal related genes*** | | | | | |
| *SCNN1B* | sodium channel, non voltage gated 1 beta subunit | 3.06 | 3.23 | 1.02E-05 | 1.14E-05 |
| *RAPGEF3* | Rap guanine nucleotide exchange factor 3 | 1.66 | 2.29 | 0.0004 | 9.50E-06 |
| *CREB3L1* | cAMP responsive element binding protein 3-like 1 | 1.28 | 1.78 | 0.0131 | 9.25E-05 |
| *CNBD2* | cyclic nucleotide binding domain containing 2 | 3.02 | 1.63 | 0.0006 | 0.0002 |
| *PRKACG* | protein kinase, cAMP-dependent, catalytic, gamma | 1.32 | 1.57 | 0.2144 | 0.0059 |
| *DGKD* | diacylglycerol kinase, delta 130kDa | -1.03 | 1.54 | 0.7072 | 0.0003 |
| *PPP1R1A* | protein phosphatase 1, regulatory (inhibitor) subunit 1A | 1.58 | 1.53 | 0.0117 | 0.0005 |
| ***Fatty acid metabolism related genes*** | | | | | |
| *SLC27A1* | solute carrier family 27 (fatty acid transporter), member 1 | 1.29 | 1.66 | 0.0042 | 0.0002 |
| *LCN12* | lipocalin 12 | 1.19 | 1.73 | 0.1056 | 0.0002 |
| *LCN1* | lipocalin 1 | 1.43 | 1.62 | 0.0035 | 0.0025 |
| *FABP5* | fatty acid binding protein 5 (psoriasis-associated) | -1.26 | -1.55 | 0.0677 | 0.002 |
| *FABP2* | fatty acid binding protein 2, intestinal | -1.48 | -1.63 | 0.0006 | 0.0007 |
| *FABP12* | fatty acid binding protein 12 | -1.48 | -1.63 | 0.0263 | 8.91E-05 |
